# A Sex-Specific Metabolite Identified in a Marine Invertebrate Utilizing Phosphorus-31 Nuclear Magnetic Resonance

**DOI:** 10.1371/journal.pone.0000780

**Published:** 2007-08-22

**Authors:** Robert A. Kleps, Terrell C. Myers, Romuald N. Lipcius, Thomas O. Henderson

**Affiliations:** 1 Research Resources Center, University of Illinois at Chicago, Chicago, Illinois, United States of America; 2 Department of Biochemistry and Molecular Genetics , University of Illinois at Chicago, Chicago, Illinois, United States of America; 3 Virginia Institute of Marine Science, The College of William and Mary, Gloucester Point, Virginia, United States of America; Baylor College of Medicine, United States of America

## Abstract

Hormone level differences are generally accepted as the primary cause for sexual dimorphism in animal and human development. Levels of low molecular weight metabolites also differ between men and women in circulating amino acids, lipids and carbohydrates and within brain tissue. While investigating the metabolism of blue crab tissues using Phosphorus-31 Nuclear Magnetic Resonance, we discovered that only the male blue crab (Callinectes sapidus) contained a phosphorus compound with a chemical shift well separated from the expected phosphate compounds. Spectra obtained from male gills were readily differentiated from female gill spectra. Analysis from six years of data from male and female crabs documented that the sex-specificity of this metabolite was normal for this species. Microscopic analysis of male and female gills found no differences in their gill anatomy or the presence of parasites or bacteria that might produce this phosphorus compound. Analysis of a rare gynandromorph blue crab (laterally, half male and half female) proved that this sex-specificity was an intrinsic biochemical process and was not caused by any variations in the diet or habitat of male versus female crabs. The existence of a sex-specific metabolite is a previously unrecognized, but potentially significant biochemical phenomenon. An entire enzyme system has been synthesized and activated only in one sex. Unless blue crabs are a unique species, sex-specific metabolites are likely to be present in other animals. Would the presence or absence of a sex-specific metabolite affect an animal's development, anatomy and biochemistry?

## Introduction

### Metabolite level sex differences

A metabolite is generally defined as any product of metabolism. For this paper we define a sex-specific metabolite as being synthesized by just one sex and as being a small, non-hormonal compound synthesized by inherent biochemistry. It is not simply a modification of an ingested compound. Using metabolite concentration terminology recommended by the Human Metabolome Project [1], we summarize some biochemical differences between sexes in several species. We then present background information on blue crabs and prior research conducted with them. The utility of Phosphorus-31 Nuclear Magnetic Resonance (P-31 NMR) for studying blue crab tissue is introduced. Finally, the discovery of a sex-specific metabolite is presented.

Hormones are trace metabolites occurring at concentrations less than 1 nanomolar. Hormone level differences between men and women are accepted as underlaying human sexual dimorphism and are believed to explain the preponderance of one sex over the other for numerous diseases: cardiomyopathy [2], [3], multiple sclerosis [4] and Lupus erythematosus [5]. Even susceptibility to parasites [6] can differ between men and women. Such hormonal effects commonly occur in many animal species [7]–[10].

Most metabolites occur at intermediate concentrations greater than 1 nanomolar but less than 1 micromolar. Metabolite levels in this intermediate concentration can also vary between the sexes. In humans, sex-specific variations occur in circulating lipids [11] , amino acids [12], [13] and carbohydrates [14]. Different metabolite levels also occur in the brain, being detected with Positron Emission Tomography [15], [16] and Magnetic Resonance Spectroscopy using H-1 [17] and P-31 [18].

Natural product levels in plants can also differ between the sexes, as documented in at least one dioecious plant, that is male and female flowers on separate plants [19]. Opportunities to study this phenomenon in plants are limited because only about 5% of plants are dioecious.

In some reptiles the sex of offspring is determined not by sex chromosomes, but by the ambient temperature during development [20]. Factors other than genetics can cause sex-specific differences in biochemical processes.

An excellent and complex H-1 NMR study of metabolites in rat urine employing Principal Component Analysis demonstrated differences between the sexes in metabolite levels, but the study also isolated and identified a novel aromatic metabolite found in males only [21]. This study may have documented the first excreted sex-specific metabolite.

Expression of rat liver nuclear proteins is significantly different depending upon the sex of the rat [22]. “A substantial quantity of scientific literature attests to the fact that there are marked or subtle gender differences in the functioning of a number of non-reproductive tissues in animal models and in humans.” [8].

### The blue crab

The blue crab (Callinectes sapidus) is a pugnacious, keystone predator and commercially valuable species that occurs in estuaries and coastal habitats of the Western Atlantic, Gulf of Mexico and Caribbean Sea [23]–[27]. In past decades, the blue crab supported the world's largest crab fishery [28], but in the last two decades blue crab catches have diminished significantly [29] as have spawning stocks in many locations, such as the Chesapeake Bay [30].

Blue crabs live in the brackish waters of estuarine systems that they inhabit with salt concentrations varying from fresh water to marine. They undergo approximately 17 molts to reach maturity at about 2 years of age and are omnivores, feeding upon various invertebrates including other blue crabs [24], [30]. Determining the sex of a mature blue crab is easy because females have red claw tips and their abdomen is rounded like the Capitol building dome; males have blue claw tips and their abdomen is long and slender not unlike the outline of the Washington Monument.

The blue crab has also been used as a model for various physiological processes including mineralization during molting [31], osmotic pressure regulation [32], and phospholipid synthesis [33]. Blue crabs have the lowest concentration of magnesium known in tissue[34].

### Nuclear Magnetic Resonance spectroscopy

The term “nuclide” specifies the number of protons and neutrons in an atomic nucleus. Hydrogen, deuterium and tritium are three nuclides of hydrogen having zero, one and two neutrons respectively. Nuclides having an even number of protons plus an even number of neutrons do not have a property called nuclear spin. Nuclides with any other ratio possess nuclear spin. Common nuclei such as Carbon 12 (C-12) and Oxygen 16 (O-16), having an even number of protons and neutrons, cannot be observed using NMR.

Many nuclei contain the appropriate number of protons and neutrons to have a spin of 1/2, making their signals narrow and their observation easier. Examples of spin 1/2 nuclides are common hydrogen H-1, carbon C-13, nitrogen N-15, fluorine F-19 and phosphorus P-31. Happily, these nuclides are found in most molecules of biological and medicinal interest and provide extensive information about their structure.

When placed in a magnetic field these nuclides can absorb radio frequency (RF) energy, and then release it, thus undergoing resonance. Each nuclide absorbs RF depending upon its intrinsic gyro-magnetic ratio and the strength of the magnetic field. The greater the magnetic field strength the higher the frequency of RF at which it resonates.

A measure of magnetic field strength is a “Tesla” which is roughly 20,000 times greater than the Earth's magnetic field. In a magnet with a field strength at 11.7 Tesla (∼234,000 times the Earth's field), H-1 resonate at 500 MHz, C-13 at 126 MHz, N-15 at 51 MHz, F-19 at 471 MHz and P-31 at 203 MHz. We observe nuclei in a magnet when they resonate, thus was born Nuclear Magnetic Resonance.

To observe a nuclide, the NMR spectrometer is adjusted to observe a specific frequency. The nuclide's exact resonance frequency is established by its gyro-magnetic ratio, the magnet's field strength and modified by the electronic environment surrounding the nuclide. As an example, ethanol has six hydrogens. One is bonded to oxygen. Two are in a methylene group and three are in its methyl group (HO-CH2-CH3). Each of these types of hydrogens experience different electronic environments thus produce three different groups of signals with three chemical shifts.

Chemical shift is measured in parts per million (ppm) which is a dimensionless unit commonly used to describe where an NMR signal is observed. A signal's ppm value is universal for all spectrometers regardless of their field strength and apply for the spectra they produce. The measured ppm depends on the electronic environment surrounding a nuclide and describes how far the signal is from a standard compound defined to be 0 ppm. I'm using 85% phosphoric acid as the ppm reference compound for P-31 NMR.

NMR spectra can also observe the relative amounts of nuclides present in the sample. This is done by comparing the area of each group under specific NMR conditions. NMR can determine the relative amounts of a nuclide within a single molecule and can also measure the relative concentrations of molecules in a mixture.

The normal NMR spectrum is a plot of signal amplitudes versus their ppm values. The relative amount of a signal is not set by its amplitude but rather by the area of the signal compared to other signal areas.

In a particular magnet, the signal to noise ratio in an NMR experiment depends on the inherent sensitivity of the nuclide and the natural abundance of that nuclide. H-1 is the most sensitive natural nuclide. The inherent sensitivity of C-13 compared to H-1 is 1.6%, for P-31 6.6%, but the natural abundance of C-13 is only 1.1% while P-31 is 100% in natural abundance. For observing the same amount of compound from natural sources, P-31 NMR is over 100 times as sensitive as C-13 NMR. Excellent and more thorough descriptions of NMR are available via web site [35] or book [36]

### Phosphorus-31 Nuclear Magnetic Resonance spectroscopy

P-31 NMR spectra of tissues contain far fewer signals than those found in H-1 and C-13 NMR spectra, thus P-31 spectra are more easily interpreted. Although P-31 is less sensitive than H-1, in situ its signals are not so drastically broadened and yields interpretable spectra. A P-31 NMR spectrum can be obtained from whole tissues in less than 20 minutes with signals approximately 30 Hz wide.

Earlier P-31 NMR studies of not perfused blue crab muscles were able to differentiate orthophosphate, arginine phosphate, phosphate esters and the phosphates in ATP, thus it seemed feasible to study other blue crab tissues. P-31 NMR provided a quick assessment of the types and relative amounts of small phosphorus metabolites in whole tissues without the need for extraction procedures. Extracts produced much better resolved P-31 spectra, but were not essential for determining the general types and relative quantity of phosphorus compounds present.

### The Unidentified Phosphorus Compound

We expanded the types of tissues examined and saw nothing unexpected until we examined crab gills. For gills there was an obvious difference in the spectra obtained from males and females with spectra from males having a major NMR signal with a chemical shift far down field from the usual phosphate signals. The presence of this Unidentified Phosphorus Compound (UPC) only in males was the impetus for this work.

The aim of this research was to determine if UPC was a sex-specific metabolite in blue crabs and to ascertain its structure. The absence of the UPC in females is difficult to prove, because an argument can always be made that it is present at a level below the sensitivity of the analytical technique used. We will provide strong evidence for the existence of a sex-specific metabolite naturally occurring only in male blue crabs.

## Materials and Methods

### Handling of blue crabs and their tissues

All blue crabs used in this study were handled in accordance with the University of Illinois at Chicago animal protocol guidelines. All crabs had a normal anatomy except for the gynandromorph crab described later. Crabs were purchased from commercial suppliers and arrived via ice cooled, overnight air delivery. Several crabs could be analyzed in a single day. Remaining crabs were kept in a cold room at approximately 12 degrees C in individual containers and were given a diet of fish twice weekly until sacrificed for this study.

**Figure 1:**
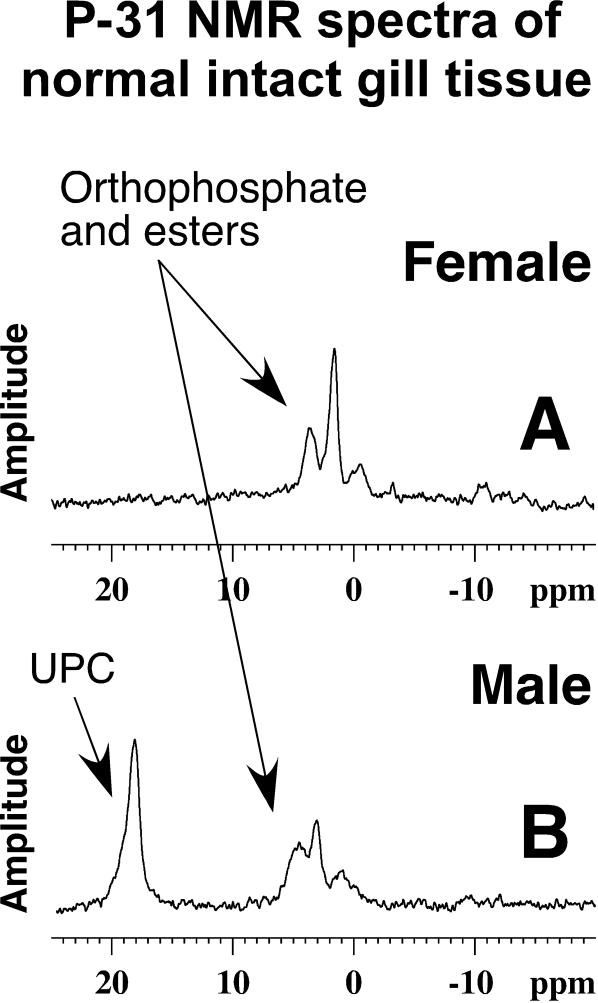
P-31 NMR spectra of male and female gills. Two P-31 NMR spectra of primary lamellae of female gill (A) and male gill (B) inserted into 5 mm NMR tubes without deuterium oxide as a “lock” solvent. Signals near 0 ppm are orthophosphate and its esters. The male's UPC is clearly separated from orthophosphates because of its significant down-field chemical shift. Signals from phospholipid and phosphorus incorporated in large polymers are extremely broadened and thus not readily observable in whole tissue. Phosphorus signals from low molecular weight compounds dissolved in tissue fluids are free to move and thus are readily observable. Typically the UPC represents a high percentage of the observable P-31 signals in males.

The crabs were ice chilled until lethargic, the carapace was opened and the following tissues were excised: gill, hepatopancreas, heart, claw muscle, leg muscle, eye stalks, hemolymph and ovaries or vas deferens plus testes. The initial surveys of whole tissues from blue crabs showed that an abundant Unidentified Phosphorus Compound (UPC) was highly concentrated in the male's gill with much smaller concentrations found in the hepatopancreas and muscle tissues. UPC was not observed in female tissues. After the correlation of UPC and the sex of the gill was initially noted, efforts focused on comparing male versus female gill tissues. Gills were excised, separated into their primary lamellae for insertion into a 5 mm NMR tube and the remaining primary lamellae frozen for later analysis. Normally gills were analyzed by P-31 NMR immediately after being excised, but frozen then thawed gills produced similar results. P-31 NMR results from male and female gill analyses are presented in Figure 1. These initial studies showing a sex specificity of UPC, provided the impetus for continuing the blue crab whole tissue research and subsequent isolation and identification of the UPC.

The gill tissue was analyzed using a Bruker AVANCE 360 MHz NMR spectrometer having a QNP 5 mm probe producing the H-1, C-13 and P-31 spectra at 360.13, 90.55 and 145.78 MHz respectively. P-31 NMR employed broad banded H-1 decoupling at room temperature without a lock solvent such as deuterium oxide being added to the NMR tube. Normally analyses took 20 minutes. Figure 1 shows P-31 spectra of primary lamella from a female and male gill. These spectra contained signals in the expected phosphate ester region at approximately 2 ppm, but male spectra routinely displayed high concentrations of the UPC at approximately 18 ppm.

### Isolation and analysis of the UPC

To identify the UPC, P-31 NMR was used to determine the types of phosphorus compounds present at each step of the isolation. The UPC extraction procedure from gills used approximately 2 g of tissue vigorously mixed in a laboratory blender with 60 ml of organic solution (chloroform∶methanol, 2∶1) for at least 5 minutes. The slurry was filtered through Whatman #1 filter paper under vacuum to hold the caked solid and pass the lipid extract that was flash evaporated, dissolved in 1 ml (deutero-chloroform∶methanol, 2∶1), analyzed by NMR and called the lipid fraction (LF)

The caked solid plus filter was vigorously mixed in a laboratory blender with 40 ml de-ionized water for at least 5 minutes. The slurry was filtered through glass wool on top of Whatman #1 filter paper under vacuum to capture the remaining residue on the glass wool. The filtrate was flash evaporated 3 times from water then dissolved in 1 ml D2O and labeled as the aqueous low MW fraction (LOMWF).

The remaining residue was digested using concentrated hydrochloric acid for 24 hours at 104 degree C to create a black slurry which was filtered, yielding the high molecular weight fraction (HIMWF). This procedure hydrolyzed all phosphorus anhydride bonds and almost all phosphate ester compounds to orthophosphate, but does not break the carbon phosphorus bond.

**Figure 2:**
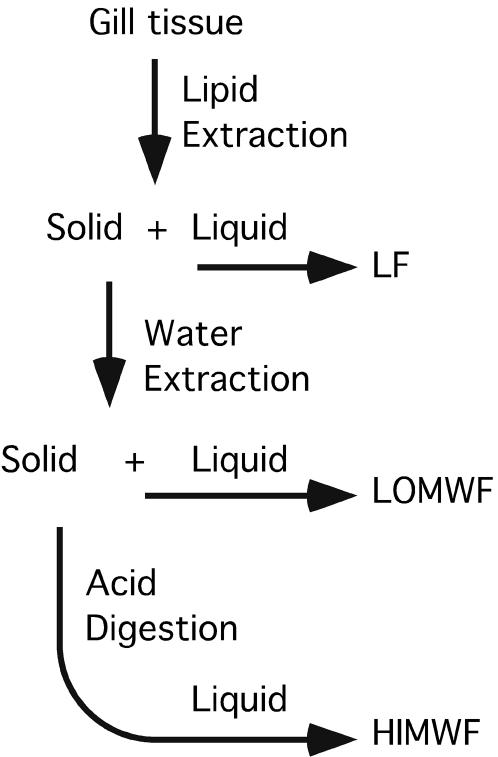
Flow diagram of gill extraction procedure. Flow diagram of gill tissue fractionation using vigorous blending with the solvent (chloroform∶methanol 2∶1) to extract the Lipid Fraction (LF). The remaining solid was then vigorously blended with water to extract the Low Molecular Weight Fraction (LOMWF). The remaining solid was digested with concentrated hydrochloric acid at 104 degrees C for 24 hours to hydrolyze any phosphorus incorporated into high molecular weight polymers. This hydrolysis yields small soluble phosphorus compounds observable by P-31 NMR. Any AEP incorporated into polymers would be released as free AEP. Free AEP is not effected by this drastic hydrolysis procedure.

For male gills, P-31 NMR analysis showed that the LF yielded several phospholipid signals. The vast majority of UPC was found in the LOMWF with trace amounts in the LF. The HIMWF had just orthophosphate and small amounts of phosphate esters. Figure 2 shows the flow diagram for the gill metabolite fractionation.

The UPC in the low molecular weight fraction was further purified. Any solutions containing particles were filtered through 0.22 um filters from Millipore corporation or were centrifuged at 13,000 rpm for 8 minutes before NMR analysis. The purification results were followed by P-31 and H-1 NMR analysis.

The LOMWF containing the UPC was passed through a Accubond II AMINO form solid phase extraction cartridge (100 mg) (Agilent Technologies) which captured virtually all phosphate compounds, but allowed the UPC through the cartridge. The filtrate still contained many non-phosphorus compounds, as evidenced by H-1 NMR analysis. In order to isolate the UPC, a 0.5 ml aliquot of the solution was applied to 2 ml of dry silica gel (Whatman bulk microparticle media 70–230 mesh) contained in a 12 ml Bio-Rad poly-prep chromatography column. The UPC loaded silica was dried by drawing air through the column using a water aspirator. The silica was washed with 10 ml of analytical grade methanol with the UPC remaining on the silica. The methanol eluted almost all non-phosphorus compounds. Finally the UPC was eluted by washing with 10 ml water, concentrated by flash evaporation, flashed 2 times with water to reduce residual methanol and dissolved in 1 ml of D2O.

**Figure 3:**
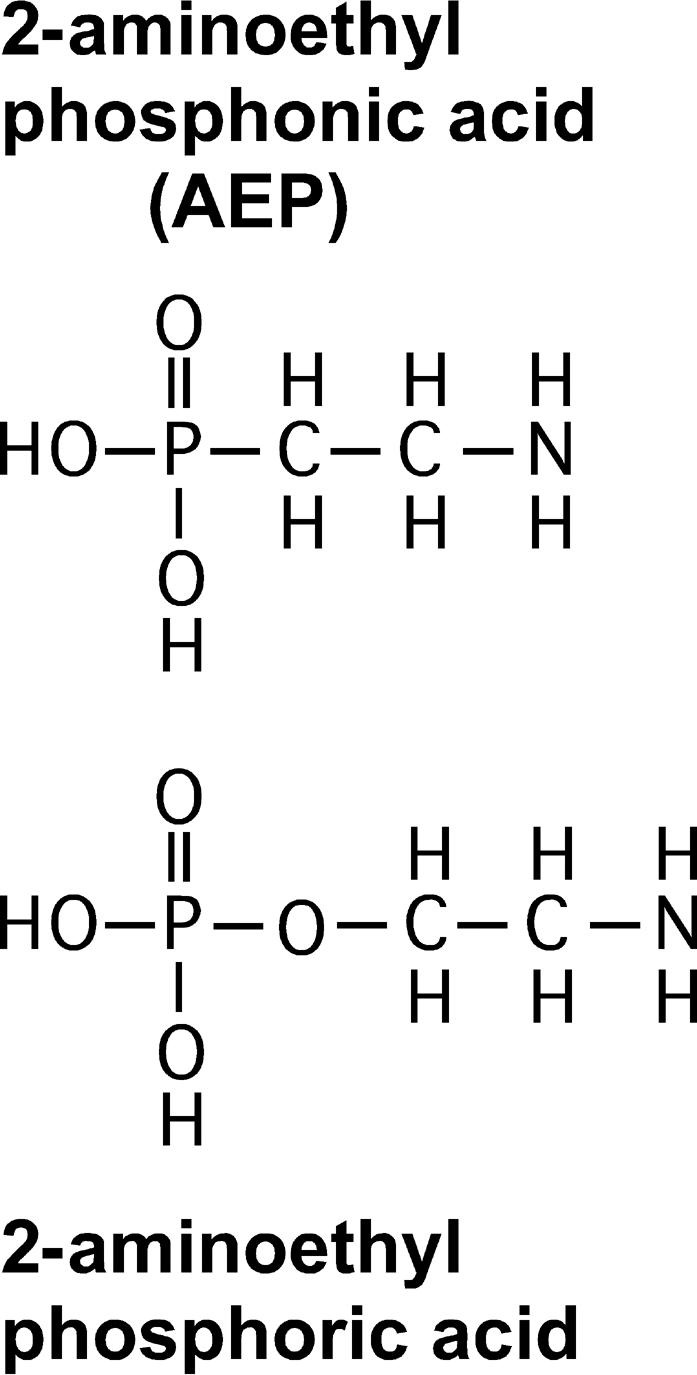
Drawing of 2-aminoethyl phosphonic acid (AEP) and 2-aminoethyl phosphate. 2-aminoethyl phosphonate (AEP), also known as ciliatine is a phosphonic acid analog of common 2-aminoethyl phosphate. AEP can be incorporated into lipids and polymers in place of 2-aminoethyl phosphate.

The purified UPC was analyzed using 1D and 2D P-31, C-13 and H-1 NMR techniques and determined to be 2-aminoethyl phosphonate (AEP), which is an uncommon, but well documented compound. Authentic AEP was added to the purified UPC to verify the assignment using P-31, C-13 and H-1 NMR. Small quantities of a modified AEP were also extracted and were most likely mono-methyl additions to the amino group in AEP. The 2-aminoethyl phosphonate (AEP), also known as ciliatine, is an analog of 2-aminoethylphosphate, a normal metabolite which is incorporated into many phospholipids. Figure 3 has the structure for AEP and 2-aminoethylphosphate.

All reagents were analytical grade and all water was de-ionized.

## Results

### Sex-specificity of AEP in blue crabs

To determine if the sex specific presence of AEP was an anomaly and limited to the initial group evaluated, blue crabs were surveyed in six different years using both commercial and scientific suppliers. To determine if geographic location affected this phenomenon, crabs were surveyed from both the Chesapeake Bay and later, the Gulf of Mexico coast of Florida. All the analyses produced similar results to those of the initial group sampled, confirming that the presence of AEP in males and its absence in females is the norm for blue crabs.

**Figure 4:**
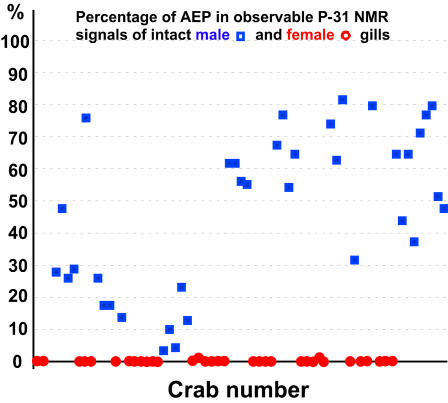
Percentage summary of AEP in male and female intact gills. A chronological presentation of results from the numerous groups of crabs covering six different years. It presents the P-31 NMR analyses of male and female intact gills with males represented by blue squares and females by red circles . The percentage of AEP for each blue crab was determined by dividing the integral of the AEP signal by the total integral from all observed P-31 signals from that crab. Only 2 of the 33 female crabs had any measurable AEP present. One of these female gills underwent a parallel fractionation with a male gill. See Figure 5.

In this paper only spectra from intermolt blue crabs were presented, but data obtained from pre-molt and post-molt blue crabs produced comparable results. Figure 4 summarizes the results from all the groups of crabs analyzed. The generally lower levels of AEP in males from Chesapeake Bay used during the earlier studies remain unexplained as do the very low levels (2% and 3%) of AEP from a single shipment of male crabs.

In whole gill surveys of females, the vast majority contained no observable AEP, but a few females exhibited less than 1% of AEP, which was attributable to cannibalism by the females upon juvenile males [24]. Female crabs could have ingested male crab tissue high in AEP, and subsequently incorporated the AEP as a phosphonate analog of 2-aminoethyl phosphate.

### Fractionation of male and female gill tissues

The fractionation procedure yielding lipid, low molecular weight aqueous and high molecular weight aqueous fractions was used for a female gill showing less than 1% levels of AEP and yielded the expected low levels of AEP. This demonstrated that there was nothing in female tissue masking the appearance of AEP in P-31 NMR spectra from female gill tissue.

In the LF from both sexes orthophosphate esters in phospholipids were present. In the HIMW fractions of both male and female gills, orthophosphate was the only major compound along with small amounts of unhydrolyzed phosphate esters.

**Figure 5:**
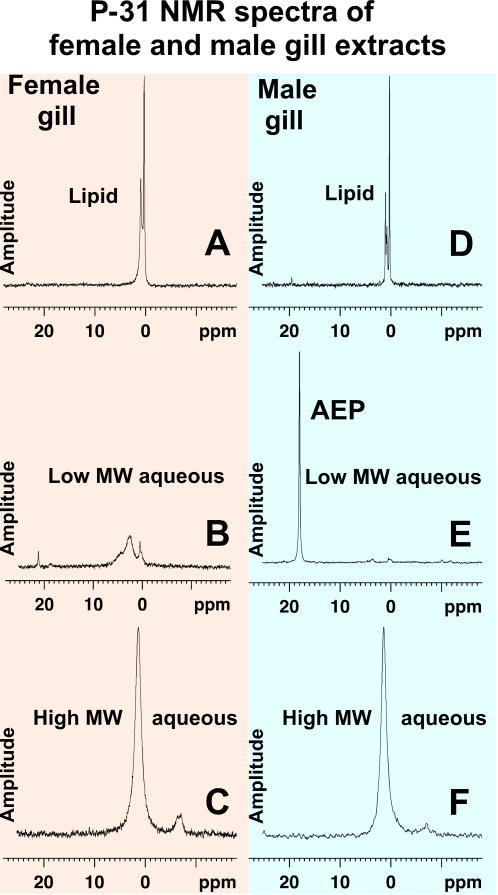
P-31 NMR spectra of female and male gill extracts. Six P-31 NMR spectra showing the three fractions A, B and C, obtained from the 1% AEP female gill and D, E and F from the male gill of Figure 1. Panels (A) and (D) present the lipid extract. Panels (B) and (E) present the low molecular weight aqueous extract. Panels (C) and (F) show small phosphorus compounds released by the acid hydrolysis of the high molecular weight aqueous fraction. The lipid extract (chloroform∶methanol 2∶1) from the female (A) and male (D) show the expected phospholipids. The low MW aqueous extraction from the female (B) has the expected orthophosphate and its esters (0 to 4 ppm) and also containing a small amount of AEP. This spectrum documents that there was nothing within intact female gill masking the appearance of AEP from P-31 NMR. The addition of sodium ethylenediaminetetraacetate, a polyvalent metallic cation chelating agent, did not alter the relative amount of AEP observed. This demonstrated that AEP was not “hidden” as a precipitate by being bound to metallic polyvalent cations. The low MW aqueous extract from the male (E) contains an extremely high percentage of AEP. Both high MW hydrolyzates show that AEP is not “hidden” by being incorporated into a high molecular weight polymer in females (C) or in males (F). This fraction is composed of orthophosphate (1 ppm) and unhydrolyzed phosphate monoesters (−6 ppm). The vast percentage of male gill AEP is present in the low molecular weight aqueous extract.

AEP was only abundant in the LOMW fraction of males. Figure 5 A,B and C are spectra resulting from the fractionation procedure for a female gill having less than 1% AEP as determined by its whole gill P-31 NMR analysis. Figure 5 D, E and F are spectra for a male gill containing approximately 50% AEP as measured in its whole gill analysis.

In order to eliminate the possibility that some factor extrinsic from gill metabolism was producing the AEP signals, gills from 2 males and 2 females were microscopically analyzed at the Shedd Aquarium, Chicago, Illinois for anatomical factors or disease conditions that could differentiate males from females, but none were found. See supporting information Figure S1. This verified that the AEP signals originated from normal gill tissue and not a microorganism or parasite in the male gills. Subsequent P-31 NMR analysis of remaining gill tissue produced spectra that were typical for their sex.

Surveys of blue crab habitats, migration routes and diets indicate that male and female crabs have similar diets and habitats [24], [26], except after mating when mature females migrate towards coastal areas to spawn and hatch their larvae [27]. Hence, it is unlikely, but still possible, that only males ingested a food yielding AEP, but the gynandromorph crab resolved this issue.

### The gynandromorph blue crab

**Figure 6:**
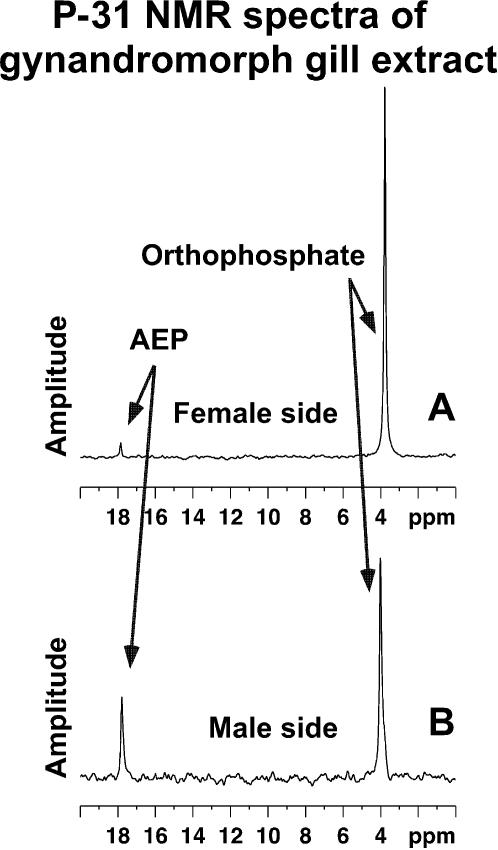
P-31 NMR spectra of aqueous extracts from the gynandromorph female and male side gills. The rare gynandromorph blue crab offered a unique opportunity to compare male and female gills from a single crab. Any differences between male and female side gills are caused by inherent biochemical differences between the sexes not a difference in the crab's diet or habitat. P-31 NMR spectra were obtained from aqueous extracts from male and female side gills, lyphilized to dryness then dissolved in a dilute solution of trisodiium ethylenediaminetetraacetate in deuterium oxide. Only two P-31 NMR signals were seen in each extract, orthophosphate and AEP. AEP on the female side A represented only 3% of the phosphorus signals. The male side gill B had 21% of its phosphorus signals in AEP. Because AEP is normally found distributed throughout the male's tissues, it was not surprising that some AEP had been incorporated into the female gill.

A rare gynandromorph blue crab, with an anatomy symmetrically and bilaterally divided between male and female, was captured in the Rappahannock River, a tributary of Chesapeake Bay. After the crab died, it was frozen. Gills were removed from the male and female side of this crab. The P-31 NMR analysis of aqueous extracts from the male and female side gills showed that the percentage of AEP found in the male side gill was over seven times greater than that found in the female side gill. Male side gills averaged 21% AEP. The female side gills averaged just 3%. See Figure 6 comparing the P-31 NMR results from the female and male side gills. These results verified that the presence of AEP in gills was not related to a crab's diet or habitat, but was inherent and dependent on the sex of the gill and its biochemistry. In normal male crabs AEP was concentrated in the gill, but it was also found in muscle and hepatopancreas tissue. It was not surprising that in the gynandromorph crab, some AEP was incorporated into the female gill as an analog of 2-aminoethylphosphate.

**Figure 7:**
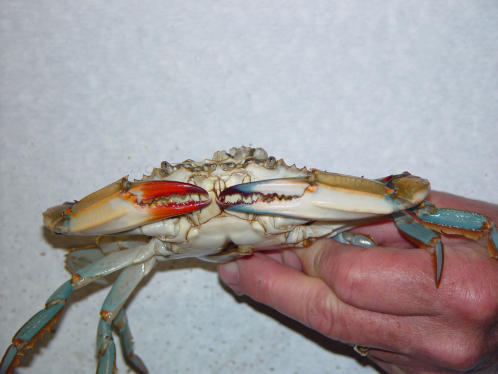
Anterior view of the gynandromorph blue crab. This an anterior photograph of the gynandromorph blue crab with the red clawed female side and the blue clawed male side.

**Figure 8:**
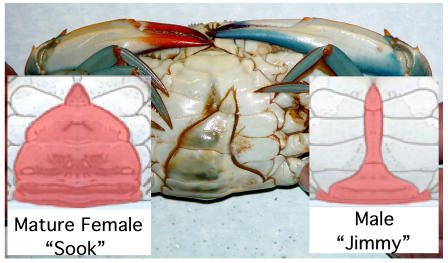
Ventral view of the gynandromorph blue crab. This ventral view of the gynandromorph blue crab shows its apron with its female side resembling the “Capitol dome” shaped apron of a normal female. The male side apron resembles the “Washington monument” shape of a normal male. The two drawing are included for comparison to normal female and male crabs.

The sexually asymmetric metabolism in the gynandromphic blue crab exactly mirrors its external anatomy. The high internal AEP level found in the male side gills reflect the external male anatomy. The low AEP levels found in the female side gills are consistent with a female incorporating AEP from a male. For an anterior view of this crab see Figure 7. The gynandromorph crab appears to be sex-specific from its sided external anatomy to its sided metabolism. The ventral view in Figure 8 presents a dramatic and clear merging of the male and female anatomies. Gynandromorphism is more commonly seen in the fruit fly (Drosophila melanogaster) than the blue crab. It seems probable that this sexual asymmetry in blue crab metabolism will also be found in fruit flies and other invertebrates exhibiting gynandromorphism.

### Statistical analysis of AEP and sex-specificity

The statistical analysis of the Figure 4 data, comparing sex of the crabs to the presence of at least 1% AEP, gave the value of calculated chi squared = 99.024 with one degree of freedom. This indicates that the crab's sex and the presence of AEP were not independent hence the crab's sex and presence of AEP were definitely related since p<0.001. The same conclusion was reached in applying Fisher's exact test.

## Discussion

### AEP, history and analysis

AEP was first identified in 1959 by Horiguchi [37] in proteolipid-like extracts of ciliates from sheep rumen. Independently, Kittredge found it in the lipid fraction from sea anemones [38]. Since then AEP has been found in many microorganisms, plants and marine animals [39]–[44]. The presence of AEP in the blue crab might have been expected because AEP does occur in other marine crabs [44].

Previously, labor-intensive chromatographic methods were needed to separate phosphonate compounds from their analogous phosphate compounds. The carbon-phosphorus bond of AEP is extremely stable to acid hydrolysis and most hydrolyzing enzyme systems. Phosphorus of this type has been classically determined indirectly, as the difference between total phosphorus and that released as orthophosphate upon prolonged acid hydrolysis. Early investigations needed to use phosphonate's stability to quantify its presence, by oxidative ashing to determine total phosphorus, followed by extensive acid hydrolysis of a second aliquot to measure the orthophosphate produced. The difference between the total phosphorus minus the acid-hydrolyzed phosphorus represented the amount of phosphonate [42].

Whole invertebrate tissues can be studied by NMR because they do not rapidly deteriorate. P-31 NMR is an efficient method for analyzing these tissues and can readily distinguish phosphonate from phosphate signals [45]. This establishes P-31 NMR as an excellent analytical technique for determining the presence of AEP in other species without extractions being necessary.

Some invertebrates synthesize AEP from orthophosphate [44], so male blue crabs may be expected to synthesize AEP and derivatize it. The function of AEP is unknown. The fact that it is concentrated in the gill may provide a usable clue. Commercially, traps are sometimes baited with live large male crabs, which attract mature females, sometimes exceeding 200 mature females per trap. Thus, AEP may be a pheromone secreted by males to attract pubescent females for mating.

### Conclusions and remaining questions

Our findings document that there is a sex-specific metabolite in blue crabs, indicating that an entire synthesizing system is being produced and activated only in males. Only because AEP was a readily discernible metabolite, was its sexual specificity obvious. If other species have sex specific metabolites, then their presence could go unrecognized in the mass of variable data obtained from H-1 and C-13 NMR spectra and from Mass Spectrometry. That AEP is specific to males was surprising. That it was found primarily in a non-sexual organ was extraordinary.

Data supporting AEP being sex-specific was obtained from P-31 NMR analysis of intact gills over several years, from fractionation and analysis of specific male and female gills and finally from a comparison of AEP results of the gynandromorph's male and female side gills. Because conservation of biological systems is common across species, it is likely that sex-specific metabolites occur in other species.

The synthetic pathway for and the function of AEP in blue crabs still need to be investigated. AEP may be a pheromone, endocrine or paracrine agent. Raising juvenile blue crabs segregated by sex then analyzing female gills for AEP, should determine if the AEP found in the two AEP containing females, was obtained through cannibalism of male crabs. Plans for testing AEP as a pheromone are ongoing.

Major questions raised by this study are: how many other species exhibit sexually specific metabolites and how would the presence or absence of these metabolites affect animal development, anatomy and biochemistry?

## Supporting Information

Figure S1 Shedd Aquarium-unpublished results
(0.19 MB TIF)
