# Supplementary figures and images for: A Sex-Specific Metabolite Identified in a Marine Invertebrate Utilizing Phosphorus-31 Nuclear Magnetic Resonance
Source: PLoS One. 2007 Aug 22;2(8):e780. doi: 10.1371/journal.pone.0000780 (PMC1940318; doi:10.1371/journal.pone.0000780)

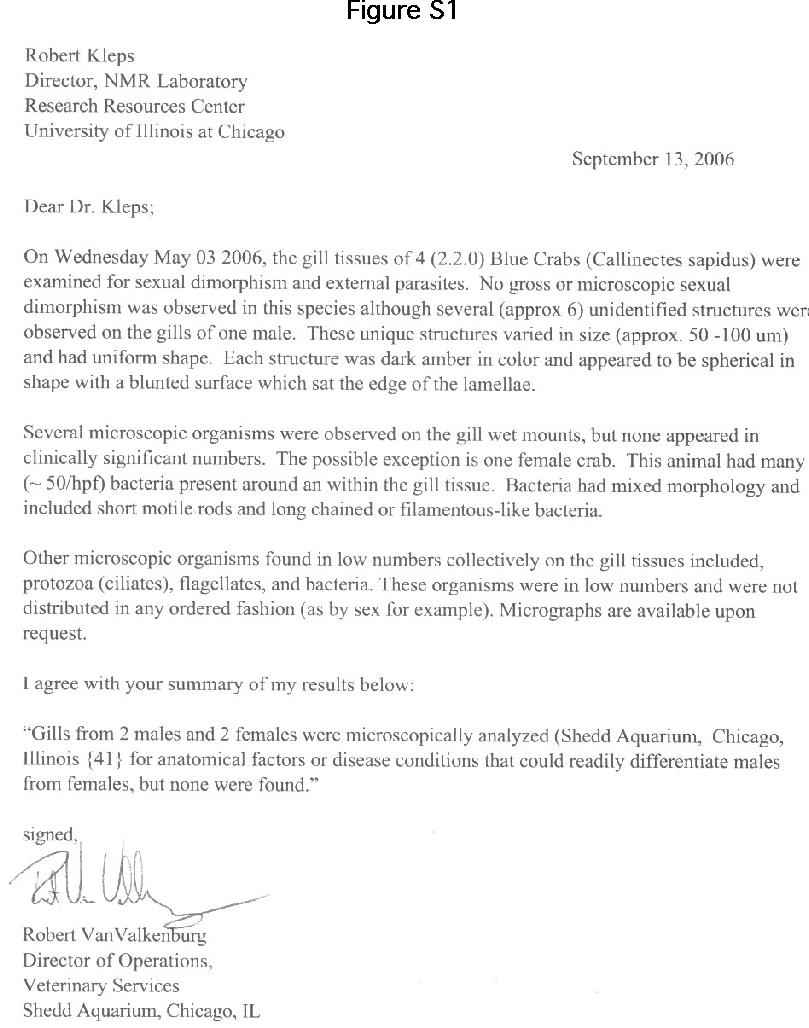

Supplement: Figure S1 — Shedd Aquarium-unpublished results (0.19 MB TIF) [file pone.0000780.s001.tif]
